# Efficacy of Sesame-Based Oil Pulling in Plaque Reduction: A Randomized Controlled Trial

**DOI:** 10.3390/healthcare13141634

**Published:** 2025-07-08

**Authors:** Christine Zürcher, Markus Nagl, Kristian Vukoje, Ingrid Heller, Sigrun Eick, Ines Kapferer-Seebacher

**Affiliations:** 1 University Hospital for Conservative Dentistry and Periodontology, Medical University of Innsbruck, 6020 Innsbruck, Austria; christine.zuercher@i-med.ac.at; 2 Institute of Hygiene and Medial Microbiology, Medical University of Innsbruck, 6020 Innsbruck, Austria; m.nagl@i-med.ac.at (M.N.); i.heller@mikrobiologie.tirol (I.H.); 3 University Hospital for Dental Prosthetics, Medical University of Innsbruck, 6020 Innsbruck, Austria; kristian.vukoje@i-med.ac.at; 4 MB-LAB-Clinical Microbiology Laboratory, 6020 Innsbruck, Austria; 5 Department of Periodontology, School of Dental Medicine, University of Bern, 3012 Bern, Switzerland; sigrun.eick@unibe.ch

**Keywords:** oil pulling, sesame oil, dental biofilm(s), oral hygiene, plaque index

## Abstract

**Objectives:** To evaluate and compare the plaque-reducing efficacy of sesame-based oil pulling versus distilled water in a randomized controlled, examiner-blinded parallel-group study. **Materials and Methods:** Forty participants with gingivitis (community periodontal index of treatment needs grade 1 or 2) were randomly assigned to either the test group (sesame-based oil) or the control group (distilled water). Participants were instructed to perform oil pulling daily in the morning for 15 min over an eight-week period. The Rustogi Modified Navy Plaque Index (RMNPI) and gingival bleeding index (GBI) were evaluated at the baseline, as well as after four and eight weeks. Additionally, biofilm samples were collected for microbiological analysis. **Results:** The RMNPI was statistically significantly reduced after eight weeks of pulling with sesame-based oil (*p* < 0.001), as well as with distilled water (*p* < 0.001), without a significant difference between the groups. The GBI was statistically significantly reduced after eight weeks of pulling with sesame-based oil (*p* < 0.002), as well as with distilled water (*p* < 0.002), without a significant difference between the groups. No significant microbiological changes were detected in biofilm samples. **Conclusions:** Both plaque and gingival indices significantly decreased with oil pulling after eight weeks of intervention. Preclinical studies are necessary to clarify the mechanism of plaque reduction by oil pulling.

## 1. Introduction

Mouth rinsing is a widely practiced oral hygiene measure aimed at preventing dental caries and periodontal diseases. The mouthwash market has experienced significant growth in recent years, primarily driven by increased consumer awareness of oral health [1]. Commonly used mouth rinsing agents include chlorhexidine, essential oil-based formulations, fluorides, and herbal extracts such as aloe vera and green tea. As an alternative to conventional mouth rinses, oil pulling is a traditional oral hygiene practice rooted in Ayurvedic medicine, in which plant-based oil is swished in the mouth to bind toxins and influence the oral microbiome. It is believed to offer various health benefits, such as reducing oral bacteria, preventing caries, combating bad breath, and supporting the body’s detoxification processes. For oil pulling, a tablespoon of oil is put in the mouth and pulled intensively between the teeth for approximately 15 to 20 min. Afterwards, the oil is expelled, the mouth is rinsed with saline or tap water, and the teeth are cleaned as usual [2,3]. While organic, cold-pressed sesame and coconut oils are traditionally preferred, other edible oils such as sunflower, olive, and rapeseed oil are also reported to be used for oil pulling. These oils contain various beneficial compounds, such as polyphenols, tocopherols, and vitamins, offering antimicrobial, antioxidant, and immunomodulatory properties [4,5]. Sesame oil, for instance, contains lignans like sesamin, sesamolin, and sesaminol, which exhibit potent antioxidative effects and boost the action of vitamins E and C, as well as provitamin A [6,7]. Coconut oil, rich in lauric acid (about 50%), is known for its antimicrobial and anti-inflammatory properties [8,9]. Olive oil, meanwhile, is a source of phytosterols, squalene, and vitamins A, E, and K, along with phenolic compounds that support antimicrobial and antioxidative functions [10,11,12]. Whether these potentially health-promoting ingredients are effective when applied by oil pulling is unclear.

Mechanical tooth cleaning remains the cornerstone of home oral hygiene, vital for preventing biofilm-induced conditions such as caries, gingivitis, and periodontitis. However, despite preventive efforts and advances in oral hygiene tools, plaque levels in the general population often remain suboptimal [13]. Research on the treatment of oral biofilm-related diseases is shifting its focus from solely biofilm-reducing methods toward modifying the oral microbiome through diet, stress management, and smoking cessation [14]. An emerging scientific field for the maintenance of oral health is natural polymers such as chitosan, which has antibacterial and mucoadhesive properties [15].

Oil pulling is believed to positively influence biofilm formation, microbial composition, and immune responses. This effect is attributed to the oil’s mechanical cleaning properties, high viscosity, saponification, and emulsification [6]. Additionally, oil creates a coating on dental surfaces, serving as a viscous barrier against plaque buildup and bacterial aggregation [16,17,18]. However, the evidence of oil pulling’s efficacy is contradictory. A recent meta-analysis of 21 randomized controlled trials comparing the effects of oil pulling to chlorhexidine or other mouthwashes found a probable benefit of oil pulling in improving gingival health [19]. However, the authors noted that the overall quality of the evidence was very low. More than half of the trials (n = 17) involved participants with no reported oral health issues, and the average study duration was just 20.7 days with a range of 7 to 45 days. In terms of biofilm composition, individual studies showed reduced bacterial counts and declines in specific microorganisms like *Streptococcus mutans* [20], *Lactobacillus species* [21], and *Candida albicans* [22] in saliva or plaque samples. Two RCTs included in a systematic review also found reductions in salivary bacterial colonies after oil pulling [23], while a meta-analysis reported significant decreases in colony counts but no substantial differences in *Streptococcus mutans* levels between oil pulling and control groups [24].

Our recent investigation into the effects of oil pulling on gingival health and biofilm formation over a period of eight weeks revealed that pulling with sesame oil significantly reduced the full mouth plaque index compared to distilled water, while no significant changes were observed in the gingival index or in the results of the microbiological analysis [25]. However, the possibility of study bias could not be ruled out, as a statistically significant difference in baseline plaque levels was observed, which may have influenced the extent of plaque reduction. Therefore, the aim of the present trial was to clarify the evidence of the plaque-reducing capacities of oil pulling and, secondly, to investigate whether a sesame-based oil with potentially effective plant additives may confer additional benefits to gingival health and the microbial composition of the biofilm. For the present investigation, a tooth oil (manufactured by Ringana GmbH, Hartberg, Austria) was selected, which also contains mint oil, curcuma extract, lemon oil, eucalyptus oil, clove oil, myrrh oil, Manuka oil, and sage oil (see Figure S4). The null hypothesis was that there is no statistically significant difference in full mouth plaque indices after eight weeks of pulling either with this sesame-based oil or distilled water.

## 2. Materials and Methods

This study received approval from the Ethics Committee of the Medical University of Innsbruck, Austria (ID EK 1117/2022), and was carried out in compliance with the 1964 Helsinki Declaration and its later revisions [26]. Written informed consent was obtained from all participants prior to their inclusion in the study.

### 2.1. Study Subjects

Forty volunteers were recruited from the Department of Dental and Oral Medicine and Cranio-maxillofacial and Oral Surgery at the Medical University of Innsbruck (Austria) between 7 February and 25 April 2023. The inclusion criteria were as follows: age ≥ 18 years, legal capacity, the presence of at least 10 teeth, and a community periodontal index of treatment needs (CPITN) score of 1 or 2 [27]. Exclusion criteria included the following: the absence of consent; a CPITN score of 0, 3, or 4; pregnancy or breastfeeding; systemic diseases or conditions that increase the risk of infection or require concurrent antibiotic therapy during dental treatment; mental and behavioural disorders affecting (verbal) communication; sesame (oil) allergy; the use of antibiotics within six months prior to or during the study period; medications that could impact gingival inflammation or bleeding (e.g., anticoagulants, cortisone); infectious diseases (e.g., HIV, hepatitis B or C); the presence of fixed orthodontic appliances; ongoing oil pulling or mouth rinsing; adult guardianship; and impaired nasal breathing.

### 2.2. Clinical Intervention

Data collection took place at the University Hospital for Conservative Dentistry and Periodontology, Medical University of Innsbruck (Austria), from 25 April to 22 June 2023.

Participants were randomly assigned to either the test group, which performed oil pulling using sesame-based oil (Ringana GmbH, Hartberg, Austria), or the control group, which used distilled water (Ampuwa Spüllösung, Fresenius Kabi AG, Bad Homburg, Germany). Randomization was performed using Microsoft Excel by generating a random number (=RAND()) for each participant, followed by sorting to allocate them evenly into study groups 1 and 2. To ensure allocation concealment, group assignments were prepared in advance and placed in sequentially numbered envelopes, which were opened only after participant enrollment.

Both liquids were provided in identical 500 mL amber glass bottles (Ringana GmbH, Hartberg, Austria). Initially, each participant was scheduled for three appointments. On the first day, participants were briefed on the study protocol, signed an informed consent form, and underwent screening to confirm their eligibility according to the inclusion and exclusion criteria. All participants were instructed to maintain their usual oral hygiene routines throughout the study. To ensure compliance, daily oral hygiene practices were recorded at the baseline and during the follow-up.

### 2.3. Baseline Investigation

All the clinical investigations were conducted by one single-blinded investigator (KV). The gingival bleeding index (GBI) was assessed dichotomously at six sites per tooth, 25 s after irritation of the gingival margin with a periodontal probe (Parodontometer PCP12, Hu-Friedy Mfg. Co., LLC, Chicago, IL, USA). Third molars, carious teeth, and implants were excluded from the assessment. The GBI was calculated as the percentage of bleeding sites relative to the total number of measured sites. The primary outcome measure, the Rustogi Modified Navy Plaque Index (RMNPI) [28], was evaluated after plaque disclosure by using 2Tone (Young, Earth City, MO, USA). The RMNPI divides the buccal and lingual surfaces into nine areas (A to I) that are scored for the presence (score = 1) or absence (score = 0) of plaque. The index assesses plaque presence across the whole mouth (areas A–I), interdental spaces (areas D and F), and gingival margins (areas A–C), offering insights into plaque distribution, as each area can be evaluated separately. Third molars and carious teeth were excluded from the evaluation, while teeth with fillings, inlays, onlays, or crowns were included. The RMNPI is calculated as the percentage of biofilm-adhering sites relative to the total measured sites.

Supragingival plaque samples were collected from one air-dried tooth (premolar or molar) in each quadrant by using a sterile Gracey curette (Kentzler-Kaschner Dental GmbH, Ellwangen, Germany) after cotton rolls were placed buccally and lingually. The tip of the curette was dipped into an Eppendorf tube containing 500 µL of sterile tryptic soy broth (TSB), and the biofilm sample was transferred into the tube by gently moving the curette for five seconds. After pooling the samples from all four quadrants, the tube was sealed and stored at 4 °C until microbiological analysis (within a maximum of 4 h).

Professional tooth cleaning was performed using an air-polishing device (Airflow^®^ Prophylaxis Master and Airflow^®^ Plus powder; both EMS, Nyon, Switzerland) and, if necessary, sonic scalers and rubber cups with polishing paste (Cleanic^®^, Kerr, Bioggio, Switzerland). The procedure was identical for both the experimental and control groups.

### 2.4. Oil Pulling

Participants were instructed to perform daily oil pulling in the early morning, immediately upon waking, on an empty stomach, and before taking any medications, practicing oral hygiene, or having breakfast. All participants were asked to rinse with their assigned liquid for a duration of eight weeks. A fixed volume of 15 mL was measured using cups (Sensoplast Packmitteltechnik GmbH, Oberhonnefeld, Germany). The liquid was swished around the mouth, pulled between the teeth, and not swallowed. After swishing the liquid throughout the oral cavity for 15 min—moving it from left to right, front to back, and vice versa, along with sucking and pulling the oil through the teeth—the liquid was spat into a waste oil container or onto a paper towel and disposed of in the trash. Following this, the mouth was rinsed gently with warm tap water to minimize the taste of the sesame-based oil, after which participants resumed their usual oral hygiene routine [29].

### 2.5. Follow-Up Investigations

Re-evaluations were performed after 28 and 56 days of oil pulling. At each visit, the same blinded investigator assessed the gingival bleeding index (GBI) and the Rustogi Modified Navy Plaque Index (RMNPI) as previously described. Supragingival plaque samples were collected from the same air-dried teeth and transferred into sterile tubes. Professional tooth cleaning was performed again at the end of the study.

### 2.6. Microbial Analysis

The protocol of microbial analysis has previously been described [25]. In brief, the tubes containing the samples in 500 µL of TSB were vortexed three times for 5 s and ultrasonicated for 2 min in a 35 kHz ultrasound water bath (Bandelin Sonorex, RK 102 H) to suspend the plaque bacteria. Afterward, the samples were vortexed again for 5 s (three times) and then diluted 1000-fold in 0.9% sodium chloride in two steps. From these dilutions, 50 µL aliquots were plated on various agar plates by using an automatic spiral plater (WASP 2; Don Whitley, Shipley, West Yorkshire, UK). The detection limit was 2 × 10^4^ CFU/mL. The selected dilution range ensured countable CFU numbers for the predominant bacterial species. Growth media included Columbia agar with 5% blood (Columbia III agar, number 254098, Becton & Dickinson), Schaedler agar (number 254084, Becton & Dickinson), chocolate agar (in-house, Institute of Hygiene and Medical Microbiology, Innsbruck), and mitis salivarius agar containing 1% potassium tellurite and 0.2 U bacitracin per ml (Sigma-Aldrich, Vienna, Austria) [30]. Plates were incubated at 37 °C, with the CFU counted after 24–72 h based on growth rates. The Columbia, chocolate, and mitis salivarius agars were incubated in 5% CO_2_, and the Schaedler agar was incubated anaerobically. The most common bacteria were identified by MALDI-TOF MS (Bruker Daltonics) using the direct smear method, with scores above 1.7 considered valid [31].

### 2.7. Statistical Methods

Sample size calculation was based on data from Zürcher et al. (2025) [25], where the same study design was used to evaluate the effect of 8-week oil pulling with sesame oil compared to distilled water with the main target parameter being RMNPI reduction. In that study, the mean RMNPI reduction was 0.2 ± 0.1 for the test group and 0.1 ± 0.1 for the control. The sample size was calculated for independent samples with a power of 80% and α = 0.05, resulting in a sample size of 17 per group. Assuming a 20% drop-out rate, the number per group was increased to 20 per group.

The microbiological findings were considered secondary endpoints, and the study was not specifically powered to detect statistically significant differences in microbiological outcomes. Sample size calculation revealed that a difference of 1 log_10_ CFU count at a standard deviation of 1 log_10_ could be detected with a power of 80% and α = 0.05 with the chosen number of participants.

The clinical data of the RMNPI and GBI were analyzed for normality of distribution by using the Shapiro–Wilk test, which indicated a non-normal distribution. Therefore, the Mann–Whitney U-test was applied to assess differences between the independent test and control groups. Pre- and post-intervention values within the same group were compared using the Wilcoxon signed-rank test, while the Kruskal–Wallis test with Dunn’s multiple comparisons was used for between-group comparisons.

To compare the progression of CFU counts of different strains between the test and control groups, the Integral Method was employed. This method converts the entire curve of CFU counts (log_10_ CFU per mL vs. time) into a single value termed “Bactericidal Activity” (BA, log_10_ CFU per mL per min) [31]. This approach allows for a more-refined statistical analysis, particularly when comparing killing curves with subtle differences. The three time points of evaluation in the study were put in the algorithm as 0, 30, and 60 min to facilitate a reliable comparison of the curves. Student’s unpaired *t*-test was applied to compare the test and control groups. The parametric evaluation arises from the method itself. For a detailed explanation of the method, please look to the original publication: Gottardi W, Pfleiderer J and Nagl M. *The Integral Method, a new approach to quantify bactericidal activity* [32].

*p*-values < 0.05 were considered statistically significant. GBI and CFU counts were secondary parameters; therefore, no adjusting for multiple testing was performed. Data analysis was conducted using SPSS software for Windows, Version 29.0.0.0 (SPSS Inc, Chicago, IL, USA), and for the evaluation of microbiological results GraphPad Prism, Version 10.2.3 (403) (GraphPad, Inc., La Jolla, CA, USA), was used. If not stated otherwise, data are given as the median and range.

## 3. Results

### 3.1. Study Population

In total, 40 individuals (25 females and 15 males; 37 Caucasians, 2 Arabs, and 1 Asian) with a mean age of 37 years (range: 23–79 years) were randomly allocated either to the control (9 females, 11 males) or test group (16 females, 4 males). There was a drop-out rate of 7.5% (two individuals in the control and one in the test group) due to non-compliance (two persons after four weeks) or antibiotic intake (one individual within the first four weeks).

### 3.2. Rustogi Modified Navy Plaque Index

**Figure 1:**
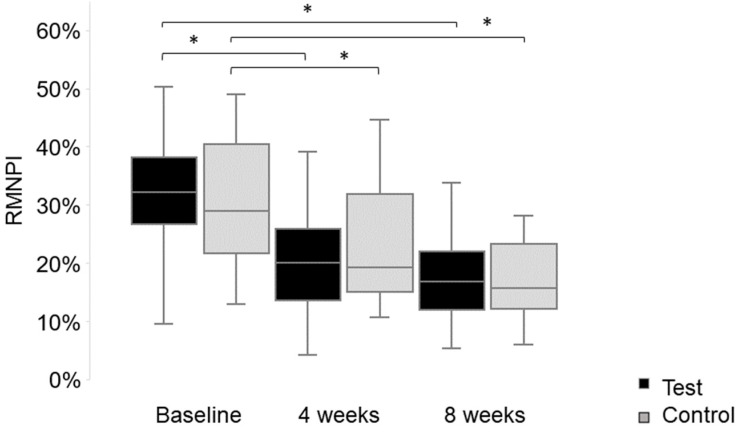
Rustogi Modified Navy Plaque Index (RMNPI; %, absolute values) at the baseline, and after 4 and 8 weeks of pulling with sesame-based oil (test group—black) or distilled water (control group—grey). The test group showed a statistically significant reduction in the RMNPI from the baseline to week four and week eight (*p* < 0.001). The same was found for the control group, with a statistically significant reduction in the RMNPI from the baseline to week four and week eight (*p* < 0.001). There was no statistically significant difference regarding the RMNPI between the test and control groups, neither at the baseline (*p* = 0.529) nor after four (*p* = 0.832) and eight weeks (*p* = 0.916) of oil pulling. The main target parameter, plaque reduction, was also not statistically significantly different (see Table 1). Statistically significant differences are marked with an asterisk.

At the baseline, the median full mouth RMNPI in the test group was 32.14% (range: 9.52–50.20%) and 28.98% (range: 12.89–49.01%) in the control group (*p* = 0.529). After four weeks of oil pulling, the full mouth reduction in the RMNPI was 10.71% (0.79–46.23%) in the test group and 11.51% (−6.55–28.60%) in the control group. Both groups showed a statistically significant reduction within the group (*p* < 0.001) (see Figure 1), but no significant difference between the groups (*p* = 0.716). After eight weeks of oil pulling, the full mouth reduction in the RMNPI was 13.99% (3.94–46.23%) in the test group and 14.25% (−0.01–28.60%) in the control group. Both groups showed a statistically significant reduction within the group (*p* < 0.001) (see Figure 1), but no significant difference between the groups (*p* = 0.987). There were no statistically significant differences between the test and control groups at any time, neither for the full mouth RMNPI nor for the subscales (see Table 1).

### 3.3. Gingival Bleeding Index (GBI)

**Figure 2:**
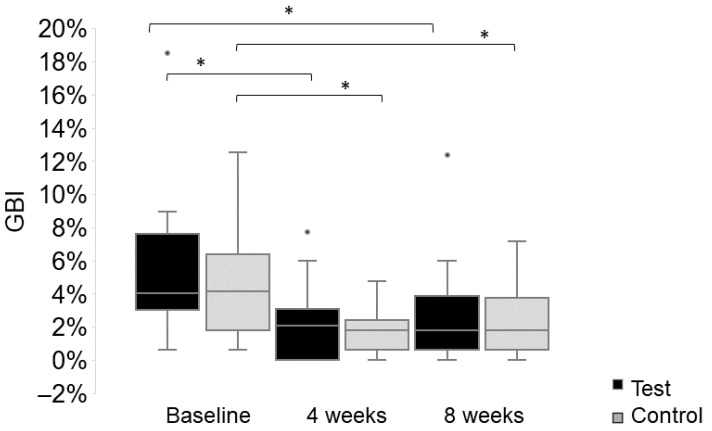
Full mouth gingival bleeding index (GBI; %) at the baseline, and after 4 and 8 weeks (absolute values) of pulling with sesame oil (test group—black) or distilled water (control group—grey). The test group showed a statistically significant reduction in the GBI from the baseline to week four (*p* < 0.001) and week eight (*p* = 0.002). The same was found for the control group, with a statistically significant reduction in the GBI from the baseline to week four (*p* = 0.001) and week eight (*p* = 0.002). There was no statistically significant difference regarding absolute levels of the GBI between the groups, neither at the baseline (*p* = 0.678), nor after four (*p* = 0.635) and eight weeks (*p* = 0.845). Statistically significant differences are marked with an asterisk.

At the baseline, the median full mouth GBI in the test group was 4.01% (range: 0.60–18.52%) and 4.17% (range: 0.60–12.5%) in the control group (*p* = 0.678). After four weeks of oil pulling, the reduction in the full mouth GBI was 1.39% (0–31.55%) in the test group and 1.92% (−1.19–10.12%) in the control group. Both groups showed a statistically significant reduction within the group (*p* < 0.001) (see Figure 2), but no significant difference between the groups (*p* = 0.536). After eight weeks of oil pulling, the reduction in the full mouth GBI was 2.38% (−1.19–31.55%) in the test group and 2.98% (−2.38–6.55%) in the control group. Both groups showed a statistically significant reduction within the group (*p* < 0.002) (see Figure 2), but no significant difference between the groups (*p* = 0.937). There were no other statistically significant differences in the subgroup analyses, neither in the test nor in the control group.

### 3.4. Microbiological Results

**Figure 3:**
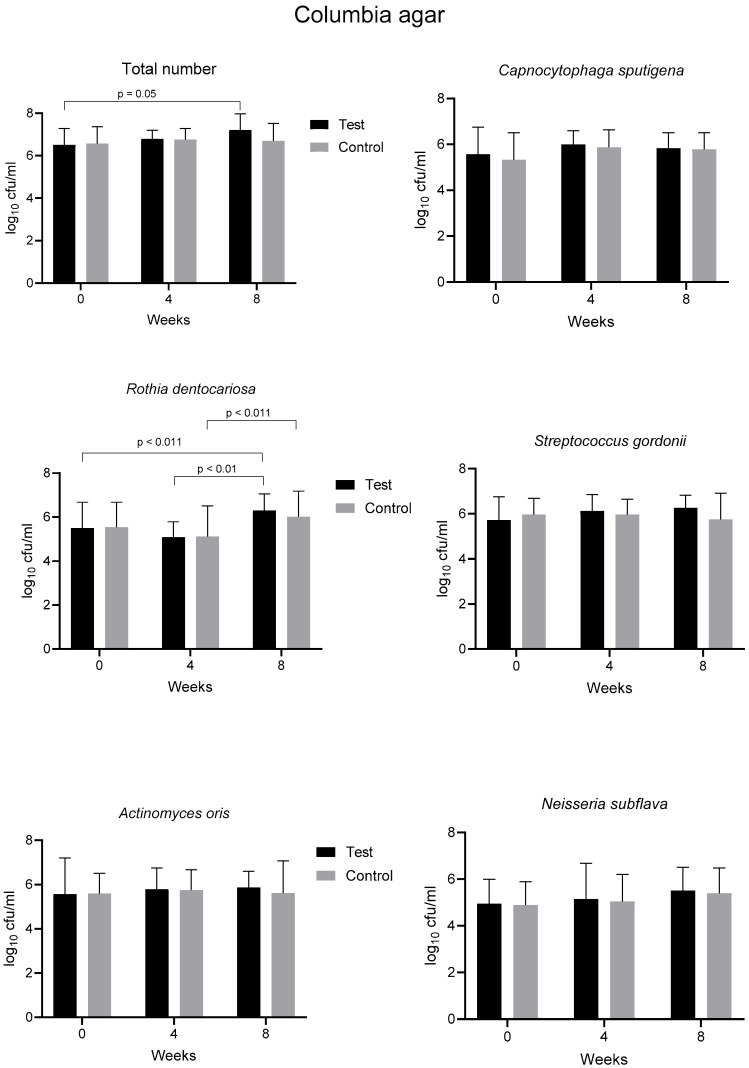
Counts of colony forming units (CFU) of bacteria grown from supragingival plaque samples on Columbia agar at the baseline and after 4 and 8 weeks of pulling with sesame-based oil (test group) or distilled water (control). The total number includes the count of all kinds of bacteria grown on the plates. Median and range of 19 test and 16 (4 weeks)–18 (8 weeks) control persons. Mann–Whitney test between pairs: *p*-values < 0.05 are indicated. Kruskal–Wallis test plus Dunn’s multiple comparison test between time points of the same group: *p*-values < 0.05 are indicated.

As in the previous study [24], we investigated the eleven prevailing bacterial species from supragingival plaque samples. Additionally, smooth and rough colonies of streptococci were distinguished by mitis salivarius agar. The courses of CFU counts of total numbers of bacteria and of numbers of single species selected for evaluation are depicted in Figure 3 and Supplementary Figures S1–S3. Overall, we could not find differences in the bacterial counts between the test and control groups, with only one exception for *Streptococcus oralis* on Schaedler agar after 1 month of pulling (difference of 0.39 log_10_, *p* = 0.0268). There were the following few scattered and small increases in bacterial counts over time within each group. These increases comprised the total number after two months compared to the baseline in the test group (0.69 log_10_ on Columbia and 0.54 log_10_ on chocolate agar), *Rothia dentocariosa* in both groups (0.81 and 0.91 log_10_ on Columbia and 1.0 and 1.14 log_10_ on chocolate agar for the test and control samples, respectively), *Streptococcus anginosus* in both groups (0.93 log_10_ and 0.83 log_10_ on chocolate agar for the test and control samples, respectively), and *Capnocytophaga sputigena* in the test group (0.90 log_10_ on Schaedler agar) (Figure 3, Figures S1 and S2). No significant changes over time were found on mitis salivarius agar (Figure S3). All significant changes remained small and within 1 log_10_ with one exception (1.14 log_10_ for *R. dentocariosa* controls on chocolate agar).

Using the Integral Method, which allows for the comparison of the courses of CFU counts over time between the groups, we found only very few significant differences. Values between the test and control groups were significantly different for *Streptococcus gordonii* (−0.005 versus 0.002, *p* = 0.018) on Columbia agar and for rough colonies of streptococci on mitis salivarius agar (−0.0076 versus −0.0001, *p* = 0.040). Remarkably, the calculations with the Integral Method confirmed the overall minimal increase in bacterial counts of the investigated species after the two months of pulling. Increases in counts were found in 87% of the total, and in 91% of the test group and 83% of the control group, with no statistical difference determined by Fisher’s exact test (*p* = 0.67). Minimal decreases in bacterial counts were only found for *S. gordonii* on Columbia agar and *Streptococcus sanguinis* on chocolate agar in controls as well as for *S. oralis* and *Leptotrichia wadei* on Schaedler ager in both groups.

## 4. Discussion

Recently, more and more patients have been enquiring about the effect of oil pulling, as it is being promoted on social media as a natural and cost-effective way to support oral health. Therefore, we investigated the effect of pulling with native sesame oil compared to distilled water. As we only found statistically significant differences between the two procedures for the RMNPI, but not for the GBI [25], we wondered whether sesame oil supplemented with natural botanical extracts and essential oils, known for its antimicrobial and anti-inflammatory properties, would have additional health-promoting effects. The formulated null hypothesis was verified through this clinical investigation. There were no statistically significant differences between the test and the control group at any time point, neither for the RMNPI, the GBI, nor microbiological analysis. However, as in the previous study, a statistically significant reduction in the RMNPI and the GBI was found for both the test and control procedures.

In the present study, sesame-based oil (kindly provided by Ringana GmbH, Hartberg, Austria) was supplemented with the natural botanical extracts of turmeric and lemon and with the essential oils of mint, sage oil, myrrh, and eucalyptus (see Figure S4). The antimicrobial [33] and clinically relevant effects of essential oil-based mouthrinses have been well documented in a systematic review on LISTERINE^®^ (Kenvue Inc., Summit, NJ, USA) [34]. Mint oil and manuka oil have been proposed as natural antimicrobial agents in mouthrinses, demonstrating efficacy without adverse effects [35,36]. Myrrh oil has been shown to exhibit multiple biological activities, including anti-inflammatory, antioxidant, and antimicrobial properties, and has proven effective in the management of gingivitis [37]. Additionally, sage, eucalyptus, and citrus oils are recognized for their antioxidant and antibacterial effects [38,39,40]. The concentrations used were within the permissible limits for cosmetic products and complied with levels considered safe.

There was an intense discussion among the authors on the control intervention. Some previous studies compared oil pulling to no intervention [41], while others used chlorhexidine [17,18,20,42] or non-chlorhexidine [43,44] mouthrinses as controls. We aimed to provide a proof of principle for any antioxidant and microbiological effects of the oil with its supplements, so we also mimicked the mechanical effect of pulling in the control group. Distilled water has no pharmacological or chemical effect, and was therefore chosen as the negative control. A double-blind study design would have required another oil as the control, which would have the same effect of emulsification and saponification. The parallel-group study design was identical to that used by Zürcher et al. 2025 [25], enabling an ideal comparison of the results between the two studies. Although a cross-over design might have provided certain advantages—such as controlling for individual variations in oral hygiene habits, adherence to oil pulling, plaque accumulation tendencies (e.g., related to tooth position), and other potential confounders—it poses a significant risk of carry-over effects. These effects arise when outcomes from the first study phase influence those of the subsequent phase, a particularly important concern in microbiological research, such as in the present paper.

As in a previous study [25], the RMNPI was significantly reduced after four and eight weeks of pulling with sesame-based oil and distilled water (both *p* < 0.001), but with no significant differences between the groups. Therefore, we hypothesize that the observed plaque reduction is mostly attributable to the mechanical effect of pulling or to a potential bias (Hawthorne effect). Participants might have improved their toothbrushing habits due to the study context, despite clear instructions given at the initial visit to refrain from making any changes. Adherence was monitored at each follow-up via structured interviews conducted by the study physician. As the patients swish the liquid around the mouth from left to right, front to back, and vice versa, along with sucking and pulling the oil through the teeth, we hypothesize two types of mechanical cleaning effects: (i) enhanced self-cleaning effect of the tongue, lips, and buccal muscles due to the swishing, and (ii) potential detaching of immature plaque due to turbulent flow. When comparing the results of the study with pure sesame oil with RMNPI levels at the baseline (41.42% [32.94–48.16]), after 4 weeks (22.82% [19.44–25.11]), and after 8 weeks (22.82% [17.86–26.74]) with the RMNPI levels in the present study using a tooth oil containing various plant oils at the baseline (32.14% [9.52–50.2]), after 4 weeks (20.03% [4.17–39.09]), and after 8 weeks (16.86% [5.36–33.76]), it can be observed that the absolute values in the current study are lower after 8 weeks of oil pulling. Thus, the additional essential oils may have had a beneficial effect on the plaque levels. However, it should be noted that the baseline RMNPI levels of the group using the sesame-based tooth oil were also lower and the reduction in the RMNPI was similar in the control group. A future trial could be valuable to directly compare oil pulling with pure sesame oil to the tooth oil over an 8-week period.

In accordance with our previous study [25], the present study demonstrated a significant reduction in the full mouth GBI in both the test and control groups (*p* < 0.002) after eight weeks of intervention, with no significant differences between the groups.

The microbiological results were consistent with those in our previous study [25]. We again could not find considerable changes in the number of the eleven investigated prevailing species in our setting over the study period. The only formally significant, but minimal difference, in the CFU count of *S. oralis* on Schaedler agar between the test and the control groups can be regarded as random; it was not found in the previous study. More remarkable is the finding of a minimal increase in CFU counts of the investigated strains over 2 months of treatment in 87% of the median values in the present study and in 74% in our previous one. This was valid for both sesame oil and distilled water. Most of the time, this observation was not significant for single strains, but overall, it indicates the absence of a bactericidal effect of the treatment, at least on the prevailing bacterial strains in vivo in our setting. It also indicates the absence of a shift in the composition of the prevailing cultivable flora. The conclusion may be drawn that pulling does not deteriorate the normal gingival microbiota. Only for *R. dentocariosa* could a significant increase in CFU counts by approximately 1 log_10_ be detected in both of our studies on Columbia agar. On chocolate agar, however, this was the case only in the present study, and it remains unclear if it is meaningful.

While the literature describes individual cases of lipoid pneumonia [43,44,45], no side effects were observed during the study duration. Neither the test group nor the control group reported cases of aspiration or accidental ingestion. There was one proband from the test group describing the urge to gag after four weeks, but this sensation was gone by the end of the study. We also monitored participants to assess their subjective perceptions of oil pulling. After eight weeks of the intervention, there were no significant differences between the groups in symptoms such as mouth dryness, reduced saliva production, sticky saliva, unpleasant taste in the mouth, and perceived bad breath. Most participants reported an improved overall mouthfeel, and nearly all expressed a desire to continue oil pulling as a regular part of their daily routine. Other unconventional methods for oral health prevention are also gaining attention, including fruit extract tablets, natural polymer rinses, and antimicrobial coatings. For example, cranberry tablets containing inactivated *Ligilactobacillus salivarius* have been shown to reduce caries progression in children [46]. Mouth rinses containing natural polymers like chitosan and propolis provide antimicrobial and anti-inflammatory benefits with improved mucosal retention. Additionally, chitosan-based coatings offer long-lasting bactericidal effects on oral surfaces and appliances [47], supporting their role as effective adjuncts to traditional oral care.

The growing number of clinical studies on oil pulling published in recent years highlights the relevance and timeliness of this topic. Patients appreciate additional oral hygiene measures they can practice at home, particularly those with health-promoting benefits. While in vivo studies provide valuable insights, they are inherently subject to bias due to variations in baseline conditions and the potential influence of the study environment itself. This remains true even when participants, as in the present study, are carefully selected and closely monitored. Several limitations of this study should be acknowledged: First, the relatively small sample size may reduce the statistical power and limit the generalizability of the findings. In future studies, larger patient groups should be used—ideally homogeneous in terms of race, while allowing for comparable distribution across genders—as this could yield more insightful and meaningful results. Additionally, the chosen control intervention represents a potential limitation, as rinsing with water may exert a mechanical cleansing effect. To better isolate this factor, future studies should consider including a third group without any intervention. Furthermore, more specific microbiological analyses—particularly targeting *Streptococcus mutans* and periodontal pathogens—could provide deeper insights into the intervention’s targeted antimicrobial effects. Only quantitatively prevailing bacterial species were investigated and estimated as representative for the evaluation of the antimicrobial effects of the treatment. Changes in species with low numbers or non-cultivable species cannot be ruled out. A clear strength of the present study is the long intervention period of 8 weeks, making it the only publication to date with such an extended observation period. Additionally, the precise patient selection is noteworthy, with a clearly defined and homogeneous diagnosis of gingivitis (CPITN grade 1 or 2). This is particularly important given that 17 of the 21 included studies in the recent meta-analysis by Jong et al. did not report a specific oral health issue of study participants [19]. Future clinical trials directly comparing oil pulling with pure sesame oil to the tooth oil over an 8-week period, in both individuals without advanced periodontal disease and those with severe periodontal conditions, would help clarify the mechanisms and efficacy of oil pulling, thereby strengthening the overall evidence base. However, there is still a significant lack of in vitro studies examining the fundamental principles of oil pulling. To establish more robust scientific evidence, in vitro investigations should explore the mechanical and therapeutic effects of various oils on biofilms.

## 5. Conclusions

Oil pulling may be considered a supplementary practice alongside mechanical dental cleaning for reducing plaque and managing gingival inflammation, and it does not adversely impact the normal gingival microbiota. However, its effects appear to result primarily from the mechanical action of swishing rather than from any specific active ingredients in the chosen oil, as the use of distilled water leads to similar results.

## Figures and Tables

**Table 1:** Rustogi Modified Navy Plaque Index (RMNPI) at the baseline, and plaque reduction after four and eight weeks of pulling with sesame oil (test) or distilled water (control). Statistically significant reductions in the RMNPI within each group after four and eight weeks of intervention compared to the baseline are marked with an asterisk. There were no statistically significant differences between the test and control group at any time, neither for the full mouth RMNPI nor for the subscales.

	Oil Pulling	Control Group	*p*-Value
**RMNPI Full Mouth**			
Baseline RMNPI, %	32.14 (9.52–50.2)	28.98 (12.9–49.01)	0.529
Plaque reduction week 4, %	10.71 (0.79–46.23) *	11.51 (−6.55–28.60) *	0.716
Plaque reduction week 8, %	13.99 (3.94–46.23) *	14.25 (−0.01–28.60) *	0.987
**RMNPI Posterior Teeth**			
Baseline RMNPI, %	35.41 (12.5–47.20)	31.94 (15.97–53.82)	0.758
Plaque reduction week 4, %	8.55 (−3.47–45.83) *	7.64 (−7.64–31.94) *	0.526
Plaque reduction week 8, %	12.39 (−0.69–45.83) *	13.54 (−3.97–31.94) *	0.657
**RMNPI Anterior Teeth**			
Baseline RMNPI, %	27.77 (2.78–54.17)	27.31 (2.31–47.69)	0.565
Plaque reduction week 4, %	12.19 (−1.85–46.76) *	12.96 (−5.09–26.85) *	0.949
Plaque reduction week 8, %	17.13 (−1.39–46.76) *	15.74 (0.93–30.09) *	0.849
**RMNPI Buccal**			
Baseline RMNPI, %	25.99 (5.16–45.73)	23.47 (9.52–51.19)	0.799
Plaque reduction week 4, %	7.82 (−4.17–45.63) *	8.33 (−10.32–41.97) *	0.437
Plaque reduction week 8, %	12.70 (−5.09–45.63) *	11.11 (−2.14–41.97) *	0.547
**RMNPI Lingual**			
Baseline RMNPI, %	35.57 (10.32–65.87)	33.73 (11.9–57.97)	0.445
Plaque reduction week 4, %	12.30 (−0.79–46.83) *	11.90 (−2.78–40.08) *	0.704
Plaque reduction week 8, %	17.86 (2.38–46.83) *	17.46 (−5.98–24.6) *	0.579
**RMNPI Approximal**			
Baseline RMNPI, %	71.91 (10.71–89.81)	62.95 (29.46–94.64)	0.314
Plaque reduction week 4, %	18.75 (−3.57–83.04) *	18.75 (−14.29–58.93) *	0.634
Plaque reduction week 8, %	15.74 (−9.82–83.04) *	25.89 (−1.92–55.56) *	0.437
**RMNPI Marginal**			
Baseline RMNPI, %	39.29 (10.12–80.13)	39.13 (13.33–77.97)	0.968
Plaque reduction week 4, %	15.48 (−2.38–62.5) *	15.48 (−11.9–41.36) *	0.579
Plaque reduction week 8, %	21.6 (5.95–62.5) *	22.62 (−5.13–46.43) *	0.975

## Data Availability

The datasets used and/or analyzed during the current study are available from the corresponding author upon reasonable request.

## Supplementary Materials

The following supporting information can be downloaded at: https://www.mdpi.com/article/10.3390/healthcare13141634/s1, Figure S1. Counts of colony forming units (CFU) of bacteria grown from supragingival plaque samples on chocolate agar at the baseline and after 4 and 8 weeks of pulling with sesame-based oil (test group) or distilled water (control). Figure S2. Counts of colony forming units (CFU) of bacteria grown from supragingival plaque samples on Schaedler agar at the baseline and after 4 and 8 weeks of pulling with sesame-based oil (test group) or distilled water (control). Figure S3. Counts of colony forming units (CFU) of bacteria grown from supragingival plaque samples on mitis salivarius agar at the baseline and after 4 and 8 weeks of pulling with sesame-based oil (test group) or distilled water (control). Figure S4: Ingredients of the sesame-based tooth oil used in this trial.

## Trial Registration

ClinicalTrials.gov NCT06841380.
