# Supplementary figures and images for: Efficacy of Sesame-Based Oil Pulling in Plaque Reduction: A Randomized Controlled Trial
Source: Healthcare (Basel). 2025 Jul 8;13(14):1634. doi: 10.3390/healthcare13141634 (PMC12294441; doi:10.3390/healthcare13141634)

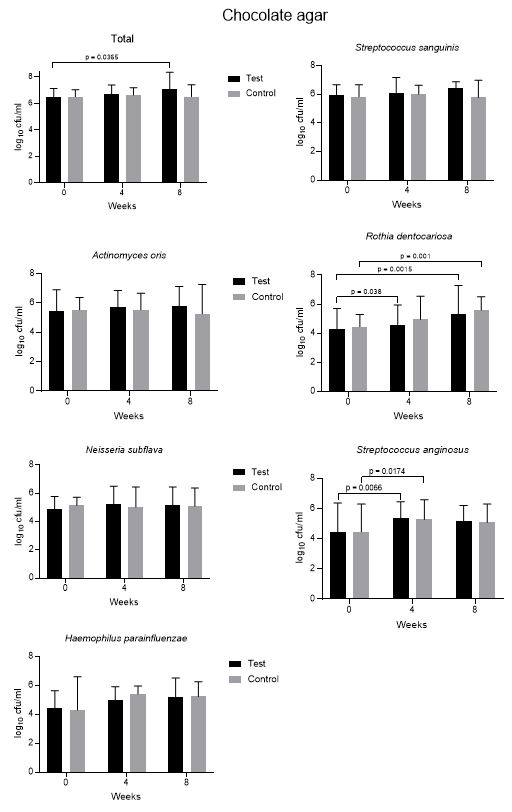

Supplement: Supplementary file 1 [file healthcare-13-01634-s001.zip › Figure S1.JPG]

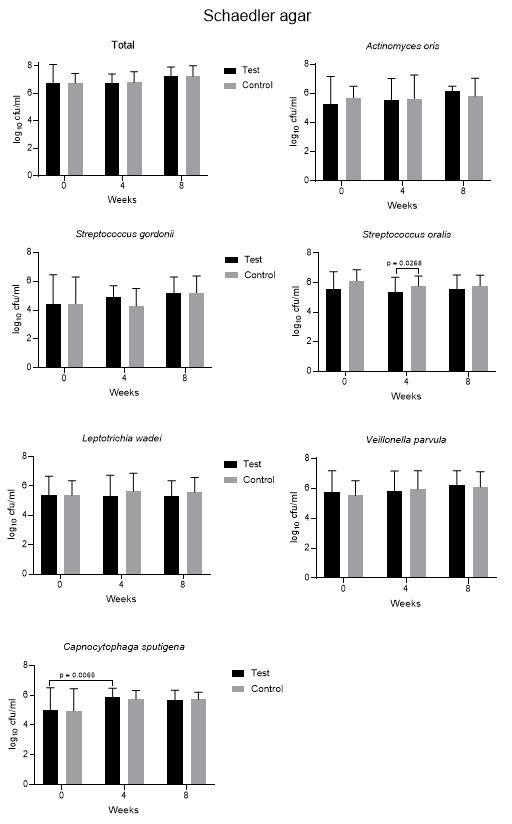

Supplement: Supplementary file 1 [file healthcare-13-01634-s001.zip › Figure S2.JPG]

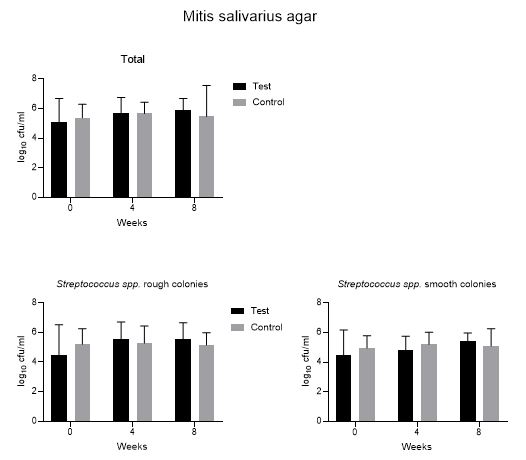

Supplement: Supplementary file 1 [file healthcare-13-01634-s001.zip › Figure S3.JPG]

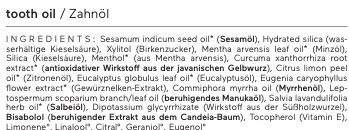

Supplement: Supplementary file 1 [file healthcare-13-01634-s001.zip › Figure S4.jpg]
